# A Rare Case of Amyloidoma of the Chest Wall Presented with Fever of Unknown Origin

**DOI:** 10.3390/diagnostics12040906

**Published:** 2022-04-06

**Authors:** Hsien-Po Huang, Shang-Feng Tsai

**Affiliations:** 1 Department of Internal Medicine, Taichung Veterans General Hospital, Taichung 40705, Taiwan; hsienpohuang@gmail.com; 2 Department of Post-Baccalaureate Medicine, College of Medicine, National Chung Hsing University, Taichung 40227, Taiwan; 3 Department of Life Science, Tunghai University, Taichung 40705, Taiwan; 4 Department of Medicine, National Yang Ming University, Taipei 11221, Taiwan; 5 Division of Nephrology, Department of Internal Medicine, Taichung Veterans General Hospital, 1650 Taiwan Boulevard Sect. 4, Taichung 40705, Taiwan

**Keywords:** amyloidoma, chest wall, fever of unknown origin (FUO)

## Abstract

Amyloidoma of the chest wall is an uncommon entity, consisting of a solitary tumor-like deposit of amyloid. Until now, while rarely reported, it was mostly presented with back pain and swelling. Here, we report the first case of a chest wall amyloidoma initially presented with fever of unknown origin. Due to the rarity of the lesion as a primary entity, protein electrophoresis and long-term follow-up are required. In addition, patients undergoing long-term hemodialysis are particularly at risk for such acquired amyloidosis. However, soft-tissue tumors, considered as amyloidoma, is also rare in patients with long-term hemodialysis. For patients with a fever of unknown primary origin, clinicians should keep amyloidoma in mind, especially in high-risk populations.

## 1. Introduction

Amyloidoma of the chest wall is an uncommon presentation of solitary tissue deposition in the absence of systemic amyloidosis. It is typically presented with back pain and swelling. So far, only seven such cases have been reported [1,2,3,4,5,6,7]. Here, we report the first case with chest wall amyloidoma initially presented with fever of unknown origin (FUO). The patient experienced FUO for several months without definite cause. We then performed single photon emission computed tomography (SPECT/CT) images through a gallium scan for a whole-body survey. A mass lesion was found over posterior chest wall. After the surgery of resection, the pathological report revealed amyloidoma. Here, we also provide a review of the literature on such amyloidoma of the chest wall.

## 2. Case Report

A 70-year-old man presented to the hospital with a complaint of intermittent fever of 38.7 °C for 3 weeks. He had also developed generalized weakness and malaise. No other localizing symptoms were found. The patient had a history of focal segmental glomerulosclerosis (FSGS), leading to an end-stage kidney disease at an early age (in his 30 s). He then started to receive peritoneal dialysis. He later received the first cadaveric renal transplant in 1987 but returned to hemodialysis in 1996 due to recurrent FSGS rated graft failure. He then received a second cadaveric renal transplant in September 2015. Additional medical history included chronic hepatitis C (without detectable virus), atrial fibrillation, parathyroidectomy in 2007 due to tertiary hyperparathyroidism, cholecystectomy in 2008 due to acute cholecystitis, bilateral total hip replacement in his 50s and, most recently, revision of right hip arthroplasty in May 2015.

He had already been hospitalized for a week prior to this admission, at which time he was treated with the empirical antibiotics Amoxicillin-clavulanate for a presumed community-acquired pneumonia, as a chest X-ray revealed increased infiltration at the right lower lobe of his lung. The patient was readmitted as he continued to develop fever despite completing the course of prescribed antibiotics.

His working profession was a teacher and had no specific contact history. There was no family or travel history of note, and he had no recent contacts with animals or with sick people. At the presentation, he was febrile (38.4 °C), with tachycardia (107/min) and hypertension (155/90 mmHg). His respiratory rate and oxygen saturation were normal. He had no rash, joint swelling, tonsillar exudate, organomegaly, heart murmur, added-on sounds upon lung examination or abdominal tenderness.

**Figure 1:**
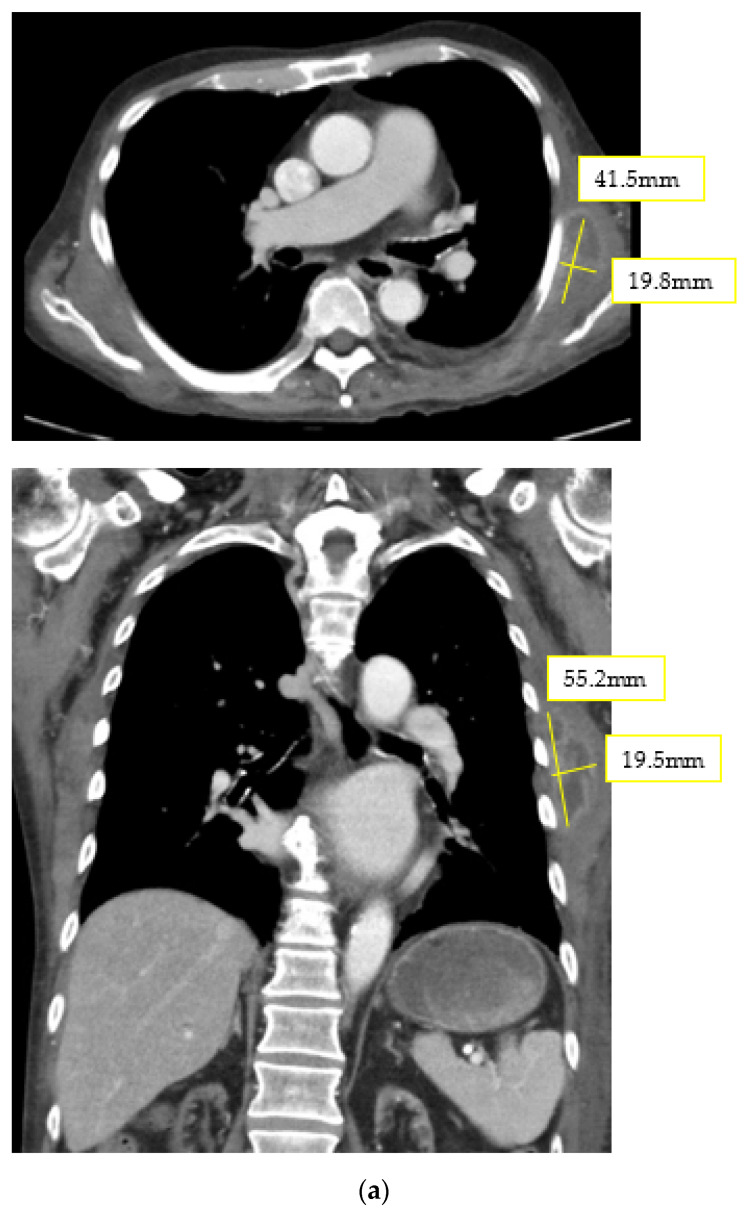

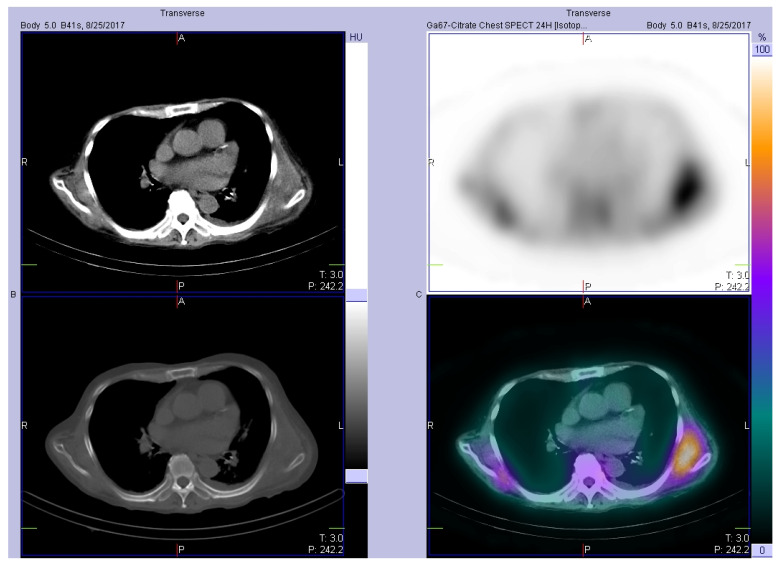

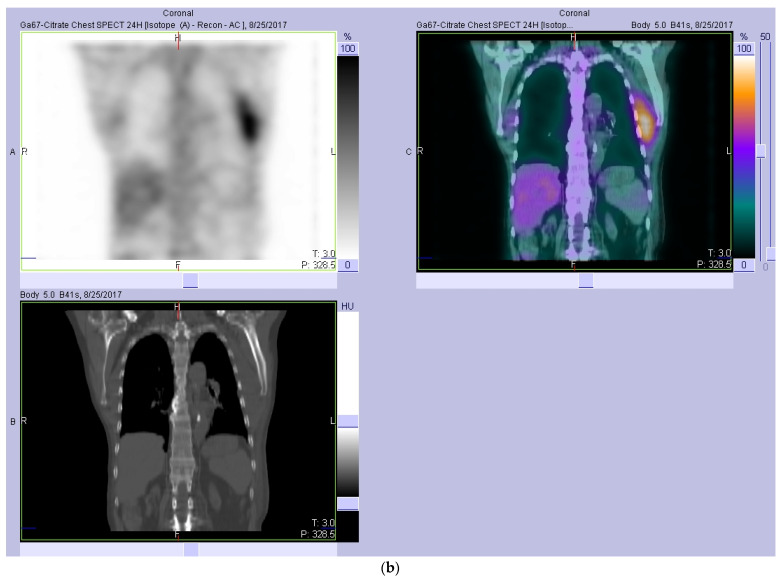
(**a**) CT scan of the chest. A heterogenous, relatively well-defined, soft tissue mass located along the left serratus anterior muscle, measuring approximately 5.5 × 4.2 × 2.0 cm^3^ in the superoinferior, oblique, and transverse dimensions. (**b**) The single photon emission computed tomography (SPECT/CT) images of a gallium scan. Note significantly greater gallium uptake at the posterolateral aspect of the left chest wall. The upper one (transverse section) and lower one (coronal section) both showed increased uptake in this mass over chest wall.

Initial blood investigations were normal apart from a normocytic normochromic anemia (8.9 g/dL of hemoglobin) and a raised level of C-reactive protein (CRP) (13 mg/dL). His blood as well as urine cultures were sterile. Serological tests were all negative for Influenza, Mycoplasma, Legionella, Cryptococcus, Chlamydia, Streptococcal, Aspergillus, Mycobacteria, Cytomegalovirus and antinuclear antibody. Two-dimensional echocardiography of the abdomen was normal. Antinuclear antibody, thyroid function and adrenal cortical function results were also negative. No apparent source of fever was detected in general checkup, and he was considered as a case of FUO. However, heart echography showed no valvular vegetation. Therefore, we ordered SPECT/CT images through a gallium scan (Figure 1b). Results showed significantly increased gallium uptake at the posterolateral aspect of the left chest wall. We then rereviewed the computed chest CT (Figure 1a), which revealed a heterogenous, relatively well-defined soft tissue mass, located along the left serratus anterior muscle, measuring approximately 5.5, 4.2 and 2.0 cm in the superoinferior, oblique and transverse dimensions, respectively.

Fine needle aspiration and trucut biopsy of the chest wall mass showed some non-representative necrotic debris, blood and histiocytes. No definite diagnosis was obtained from the biopsy. Due to diagnostic challenges, a surgical resection of the mass was carried out with an open excision. Overall, the specimen had a rubbery and tan appearance and was 5.5 × 4.2 × 2.0 cm^3^ in size. Histologic examination of the specimen was consistent with amyloidoma, with Congo red stain confirming amyloid deposits and apple-green birefringence under polarized light. The diagnosis was compatible with a β-2-microglobulin related amyloid deposition.

The patient had no postoperative complications. He did not have any fever during the week prior to discharge. The patient remained in a stable condition, with no evidence of recurrence.

## 3. Discussion

Tumor-like localized amyloid deposits (amyloidoma) are uncommon. They appear in the gastrointestinal tract, bladder, lung, head and neck region and soft tissues. Until now, only seven instances of such amyloidoma on the chest wall have been reported [1,2,3,4,5,6,7]. The differential diagnosis includes a primary tumor, metastatic disease or infectious process, and the disease is not clearly delineated prior to a tissue diagnosis [1]. Amyloidoma is considered a benign growth, since there is no evidence of it causing reduced life expectancy. Local resection results in low recurrence rates if presented early with minimal local invasion [1,2]. For lesions not amenable to resection, radiotherapy is an alternative treatment choice. Because of the rarity of the lesion as a primary entity, protein electrophoresis and long-term follow-up are necessary [1,8].

Initially, many cases of such amyloidoma appear asymptomatic. However, they likely become painful because of the growth, invasion of surrounding structures or the development of pathologic fractures. Until now, only seven cases of chest wall amyloidoma have been reported [1,2,3,4,5,6,7] (summary in Table 1). Of them, most (six cases) were presented with back pain and swelling [1,2,3,4,5,6], and one patient manifested worsening shortness of breath [7]. Our case is the eighth such case of chest wall amyloidoma. Interestingly, our case was the first that was presented with an initial fever of unknown origin. Of all eight cases of chest wall amyloidoma, the patients’ mean age was 64.8 years old, and 87.5% were male. Most cases (87.5%) received local excision, and one received conservative treatment involving pain management and close observation. Therefore, in this case, we highlighted the survey of FUO showing the amyloidoma. Meticulous physical examination and image study should be applied to detect this rare condition.

In the literature, we identified only two studies on localized amyloid deposits with FUO [8,9]. The first case was a 65-year-old woman diagnosed with primary amyloidoma of the lung parenchyma presenting with fever for 8 months, with a cough and shortness of breath. She was treated with external beam radiation and corticosteroid with palliative intent. She was leading a good quality of life after 6 months of follow-up [8]. The second case was a 43-year-old woman diagnosed with localized, insulin-induced cutaneous amyloidosis presenting with a 2-year history of intermittent fever, chills and fatigue. Because of the occasional hemorrhage and pain, the long-term plan would be to surgically remove the amyloid deposits [9]. Our patient was the third case, presenting with localized amyloid deposits with FUO. Our patient was the first case with chest wall amyloidoma.

Our patient underwent long-term renal replacement therapy (1-year peritoneal dialysis and 5-year hemodialysis). He also experienced three courses of chronic kidney disease (10 years for native kidneys, 7 years for first-time graft kidney and 5 years for the current graft kidney). Therefore, he was at extremely high risk for developing acquired amyloidosis. Dialysis-related amyloidosis (DRA) is common in patients under dialysis for extended periods. It is a disease with the characteristics of accumulation and tissue deposition of amyloid fibril, which is composed of β-2-microglobulin in the patients with renal dysfunction. Even with current modern hemodialysis (the high-flux biocompatible membrane of artificial kidneys and convective therapies), β-2-microglobulin is still retained in patients’ body due to reduced clearance. One analysis of data obtained in the past era of dialysis indicated a prevalence of virtually 100% of patients surviving over 15 to 20 years on regular dialysis treatment [6]. Beta-2-microglobulin can be detected in almost every organ, resulting in carpal tunnel syndrome, tenosynovitis, bone cyst, periarthritis and gastrointestinal tract involvement. However, an isolated amyloidoma present in individuals with ESKD on chronic hemodialysis is rare, and it most commonly occurs in the osteoarticular system. Until now, only four cases have been reported in ESKD patients with soft tissue amyloidoma [6,10,11,12]. Two cases of amyloidoma were in the buttock [10,11], one in the genital region [12] and the remaining one was in the thoracic and abdominal walls [6]. Our patient was the fifth case of soft tissue amyloidoma and the second case of chest wall amyloidoma in ESKD. This distinctive presentation could be easily missed in routine patient care.

## 4. Conclusions

Chest wall amyloidoma is currently rare, and initial FUO is an uncommon presentation. In daily routine and FUO checkup, clinicians should keep this rare diagnosis in mind for timely treatment.

## Figures and Tables

**Table 1:** Reported cases of chest wall amyloidoma.

Case, Year	Age, Gender	Medical History	Presentation	Treatment, Outcome
Chest wall amyloidoma, *n* = 8				
1 [1], 1995	53, M	Unremarkable	Radicular pain, numbness of both lower extremities	Wide local excision, with no recurrence
2 [2], 2008	75, M	Left thoracoplasty due to tuberculosis at age of 20	Thoracic pain, localized to the left shoulder	Wide local excision, with no recurrence
3 [3], 2012	65, F	Unremarkable	Increasing lump in left lower chest	Wide local excision, with no recurrence
4 [4], 2013	62, M	Myocardial infarction at age of 50, prostatectomy for prostate cancer at age of 55	Back pain and swelling	Radiotherapy and resection of the right lower lobe, chest wall, diaphragm, and T9 *, with no recurrence
5 [5], 2016	77, M	Unremarkable	Nodular swelling over right chest	Wide local excision, with no recurrence
6 [6], 2020	55, M	ADPKD related ESRD, received renal transplant at age of 25, but returned to HD for 30 years due to rejection	Left lower abdominal pain and fullness	Conservative treatment involving pain management and close observation
7 [7], 2021	61, M	Right apical lung plasmacytoma at age of 50	Worsening shortness of breath	Wide local excision, with no recurrence
8, 2017 (This case)	70, M	FSGS related ESRD, received 1st renal transplant at age of 40, graft failure at age of 49, and received 2nd renal transplant at age of 68	Fever of unknown origin	Wide local excision, with no recurrence
Subtotal	64.8, M (87.5%)			Surgery: 87.5% Conservative treatment: 12.5%

* Complicated with T9 plasmacytoma.

## Data Availability

Not applicable.
